# Effects of single bout exercise with different cognitive demands on inhibitory control in elementary school children

**DOI:** 10.3389/fpsyg.2026.1879513

**Published:** 2026-10-07

**Authors:** Chen Chang, Chiung-Ling Chu, Yi-Lin Kuo, Ting-Yu Chueh, Tsung-Min Hung

**Affiliations:** 1 Department of Physical Education and Sports Sciences, National Taiwan Normal University, Taipei, Taiwan; 2 Master’s Program of Transition and Leisure Education for Individuals with Disabilities, University of Taipei, Taipei, Taiwan; 3 Institute for Research Excellence in Learning Science, National Taiwan Normal University, Taipei, Taiwan; 4 Lifestyles of Health and Sustainability Executive Master of Business Administration, National Taiwan Normal University, Taipei, Taiwan

**Keywords:** children, cognitively demanding, distributional analysis, exercise, inhibitory control

## Abstract

**Introduction:**

Inhibitory control is a core component of executive function that develops substantially during childhood. Although cognitively demanding exercise has been proposed to enhance inhibitory control, findings regarding the effects of varying cognitive demand remain inconsistent. This study examined the acute effects of exercises with different cognitive demands on inhibitory control in children.

**Methods:**

Twenty-six children (Mage = 11.8, SD = 0.5) completed three exercise conditions in a randomized crossover design: high cognitively demanding exercise (HE), low cognitively demanding exercise (LE), and active control (AC). Inhibitory control was assessed using the Flanker task. Change scores in reaction time, accuracy, and Flanker effect were analyzed, and exploratory distributional analyses were conducted using conditional accuracy functions and delta plots.

**Results:**

Reaction time and accuracy did not differ across exercise conditions (all *p* > 0.05). However, the reduction in Flanker effect was greater following AC than LE (*p* = 0.04, *d* = −0.81). No statistically significant condition-related differences were detected in the exploratory distributional indices (all *p* > 0.05); these analyses should be interpreted as hypothesis-generating.

**Discussion:**

Overall, these findings suggest that exercises with higher cognitive demand may not yield greater benefits to inhibitory control than lower-demanding exercises under the higher-than-intended physical intensity elicited in the present protocol. Future studies should consider children’s limited physical and cognitive resources when designing interventions to optimize the potential synergistic effects of cognitively demanding exercise.

## 1 Introduction

Inhibitory control (IC), a core component of executive function (EF), refers to the ability to regulate attention, behavior, thoughts, and emotions to override strong internal predispositions or external lures (Diamond, 2013). IC prospectively predicts a wide range of long-term outcomes in adulthood, including physical health, substance dependence, financial well-being, and criminal behavior (Moffitt et al., 2011). Latent network analysis revealed that IC-related tasks exhibited greater centrality, suggesting a pivotal role in organizing and supporting other EF processes (Menu et al., 2024). Developmental research indicates that IC undergoes rapid development during early childhood, with relatively smaller changes observed into adolescence (Best and Miller, 2010; Best et al., 2009; Petersen et al., 2016; Tervo-Clemmens et al., 2023; Williams et al., 1999). Accordingly, interventions that strengthen IC during late childhood are developmentally sensitive and may yield lasting cognitive and behavioral benefits.

Meta-analyses suggested that acute exercise can improve IC performance in children (Álvarez-Bueno et al., 2017; Oberste et al., 2019; Verburgh et al., 2014), and that exercise involving higher cognitive demands tends to produce stronger effects on IC than less complex modalities (Chu et al., 2024; Formenti et al., 2021; Gutiérrez-Capote et al., 2024; Mao et al., 2024; Singh et al., 2025; Wang et al., 2024). These findings were consistent with the cognitive stimulation hypothesis (Best, 2010; Pesce, 2012), which posits that activities enriched with cognitive challenges confer greater benefits than purely aerobic exercise. Such cognitively enriched exercise may activate cerebral regions responsible for the regulation of goal-directed behaviors, thereby supporting improvements in EF (Best, 2010; Diamond, 2000; Pesce, 2012; Tomporowski and Pesce, 2019). Extending this perspective, the “Thinking while moving” framework (Herold et al., 2018) proposes integrating cognitive tasks directly into motor training. This integrated approach has been shown to induce greater cognitive benefits than sequential motor-cognitive training (Fissler et al., 2013; Gavelin et al., 2021; Tait et al., 2017).

Despite the promising findings, several factors need to be considered to achieve the expected cognitive benefits of cognitively demanding exercise. First, effective cognitive enhancement depends on the alignment between the cognitive processes engaged during exercise and those assessed in subsequent tasks (Bedard et al., 2021; Bulten et al., 2022; Schmidt et al., 2016; Tomporowski and Qazi, 2020; Vazou and Smiley-Oyen, 2014). Second, exercise of adequate duration and moderate intensity has been shown to facilitate attention-related performance in children (Anzeneder et al., 2023b; Budde et al., 2008). In contrast, high-intensity exercise (Jäger et al., 2015; Schmidt et al., 2016) or prolonged exercise (Bedard et al., 2021; Bulten et al., 2022; Egger et al., 2018; Gallotta et al., 2015; Gallotta et al., 2012) may deplete attentional and inhibitory control resources, thereby hindering observable differences between exercise conditions with high and low cognitive demands.

Although accumulating evidence suggests potential benefits of cognitively engaging exercise, most previous research has focused on comparing exercise conditions with and without cognitive demand. In contrast, evidence directly comparing exercise with high versus low levels of cognitive demand remains limited and inconsistent (Cabral et al., 2026; Paschen et al., 2019). For example, Anzeneder et al. (2023a) found that children performed better on an attentional task after exercise with high cognitive demand than exercise with low or moderate cognitive demand, whereas Ludyga et al. (2024) observed greater improvements in the affective Stroop task following exercise with low rather than high cognitive demand. Therefore, these mixed findings indicate that the effects of varying levels of cognitive demand remain unknown.

Most studies examining exercise-cognition relationships have relied on mean reaction time and overall accuracy as the primary outcome measures. However, these aggregate measures may fail to capture differences in speed–accuracy trade-offs (SAT) or response strategies (Draheim et al., 2019; Heitz, 2014). Accordingly, researchers have recommended the adoption of accuracy-based metrics and distributional methods to provide more informative assessments of executive processes in intervention research (Draheim et al., 2019; Heitz, 2014). Despite these advances, distributional analyses have primarily been applied to examine cognitive modulation during exercise (Davranche et al., 2009; Davranche and McMorris, 2009; Schmit et al., 2015) but have rarely been used to characterize post-exercise changes in inhibitory control.

Two complementary approaches are commonly applied to examine the dynamics of activation and suppression processes underlying IC (Ridderinkhof, 2002). The conditional accuracy function (CAF) plots response accuracy across reaction time bins, illustrating how accuracy changes from fast to slow responses (Gratton et al., 1992; Ridderinkhof, 2002). The delta plot (DP) depicts the magnitude of the interference effect across the reaction time distribution, supporting inferences about the evolving balance between automatic activation and top-down inhibition (De Jong et al., 1994). Within the activation–suppression framework, early positive slopes reflect strong automatic response activation, whereas later flattening or reversal indicates the gradual engagement of inhibitory control. Together, combining CAF and DP provides a process-level view of inhibitory control by showing how conflict monitoring and suppression unfold across the response distribution (Gratton et al., 1992; Ridderinkhof, 2002; Ulrich et al., 2015).

In summary, the present study aimed to investigate the effects of exercise with different levels of cognitive demand on inhibitory control in children. The intervention protocol proposed by Chueh et al. (2023) was adopted and adapted, as it has been shown to impose different cognitive demands and to facilitate neural processes related to stimulus evaluation in young adults. Furthermore, in addition to the conventional aggregate indices (mean reaction time, accuracy, and difference scores), CAF and DP were conducted as exploratory secondary analyses to characterize behavioral patterns across the reaction-time distribution. Based on the prior findings, we hypothesized that HE would produce greater pre-to-post improvements in Flanker task performance than LE and AC, reflected in (H1) larger reductions in mean reaction time and Flanker effect and (H2) larger increases in mean accuracy. Exploratory secondary analyses using the conditional accuracy function (CAF) and delta plot (DP) were conducted to characterize condition-related patterns across the reaction-time distribution. No directional hypothesis was specified for these analyses.

## 2 Materials and methods

### 2.1 Sample size estimation and justification

*A priori* sample size was determined based on a previous study reporting a moderate effect of acute cognitively demanding exercise on inhibitory-control reaction time in school-aged children (Cohen’s *d* = 0.63; Ishihara et al., 2017). Accordingly, a medium effect size (Cohen’s *f* = 0.25) was selected as the smallest effect size of interest. The power analysis was performed using G*Power (version 3.1.9.6) (Faul et al., 2007), indicating that a sample of 24 participants would be sufficient to achieve 80% power with an alpha level of 0.017 (adjusted for multiple pairwise comparisons). Although the dropout rate in similar school-based interventions has been relatively low (Bedard et al., 2021; Bulten et al., 2022), we anticipated a 20% dropout rate resulting in a total planned sample size of 30 participants.

### 2.2 Participants

To ensure safety in interval training among school-aged children (Liu et al., 2024), the present study recruited track and field team members who regularly participate in at least 150 min of moderate to vigorous physical activity per week. The inclusion criteria were as follows: (1) aged between 10 and 12 years and (2) normal or corrected-to-normal vision. The participants were excluded if they (1) had a body mass index (BMI) of 27 kg/m^2^ or higher, (2) were not right-handed, or (3) had any diagnosed disorders according to the International Classification of Functioning, Disability and Health. Initially, thirty elementary school students in Xindian District, New Taipei City were recruited; four withdrew due to personal reasons, resulting in a final sample of 26 participants.

### 2.3 Measurements

#### 2.3.1 Cognitive performance

Inhibitory control was evaluated utilizing a modified version of the Eriksen Flanker task to assess inhibitory control, programmed using E-Prime 3.0 (Psychology Software Tools, Inc., Sharpsburg, PA). Participants were presented with an array of white arrows against a black background at the center of a laptop monitor. The stimuli consisted of either congruent (< < < < < or > > > > >) or incongruent (< < > < < or > > < > >) configurations based on the direction of the center arrow. The ratio of congruent to incongruent stimuli was two to one. Participants were instructed to respond by pressing either button “1” (left-pointing arrow) or button “3” (right-pointing arrow) upon the onset of a stimulus.

During the task, participants first observed a white fixation cross at the center of the monitor for 1,000 ms, after which they were required to respond as quickly and accurately as possible within the 1,000-ms stimulus presentation time. The inter-stimulus interval (ISI) ranged between 1,300 and 1,500 ms to prevent anticipation by participants that could affect the results. Participants were required to score above 80% in the 20 practice trials to ensure their familiarity with the task before proceeding to the formal trials. A total of 216 trials were divided into three blocks, with a 1-min rest period between blocks.

#### 2.3.2 Felt Arousal Scale and Feeling Scale

The Felt Arousal Scale (FAS) and the Feeling Scale (FS) were employed as subjective measures of an individual’s arousal and affective valence. The FAS serves as a single-item indicator of activation on a 6-point scale, ranging from 1 (low arousal) to 6 (high arousal) (Svebak and Murgatroyd, 1985). The FS functions as a single-item indicator of affective valence on an 11-point scale, spanning from −5 (very bad) to +5 (very good; Hardy and Rejeski, 1989).

#### 2.3.3 Ratings of perceived exertion (RPE) scale

The walking/running evaluation version of the OMNI Scale for children was used to measure exercise intensity in this study (Utter et al., 2002). The OMNI Rating has been validated as a subjective measurement of an individual’s perceptions of physical effort (Borg, 1982; Robertson et al., 2003). The scale is an 11-point scale ranging from 0 (not tired at all) to 10 (very, very tired), depicted with a pictorial description of children walking/running up an incline. A rating of 7 to 8 points on the OMNI Scale indicates moderate-to-vigorous intensity exercise, which was the targeted score for the intervention in this study.

#### 2.3.4 Heart rate monitor

Heart rate (HR) represents the activation of the sympathetic nervous system (Grassi et al., 1998) and was regarded as the index of exercise intensity (Garber et al., 2011). The heart rate data were collected from 14 participants who agreed to wear the heart rate monitor. The same participants wore the monitor (Polar RS800CX; Polar Electro Oy) in all three experimental conditions. HR data were recorded at the following time points: (1) 5 min before the pre-test; (2) during the pre-test; (3) during the intervention; and (4) during the post-test. The exercise intensity was targeted at 65% ~ 75% of an individual’s maximal heart rate, estimated using the formula 208 – (0.7 * age) (Mahon et al., 2010).

#### 2.3.5 Cognitive demand

The Mental Effort Rating Scale (MERS) (Paas, 1992) is a 9-point Likert scale that ranges from 1 (very low mental effort) to 9 (very high mental effort), used to subjectively measure the mental effort exerted by an individual during an intervention.

#### 2.3.6 Procedure

This study adopted a randomized crossover design, with each participant taking part in three separate sessions, which were high cognitively demanding exercise (HE), low cognitively demanding exercise (LE), and active control (AC). To minimize any order or learning effects, the experimental sessions were counterbalanced across six sequences of experimental conditions (Figure 1). Prior to participation, all children were provided with age-appropriate information regarding the study’s purpose and procedures. Written informed consent was obtained from their parents or legal guardians in accordance with ethical guidelines for research involving minors. During the familiarization session, participants underwent an eligibility assessment, received a detailed explanation, and were acquainted with the various measurements used in the experimental procedures.

**Figure 1:**
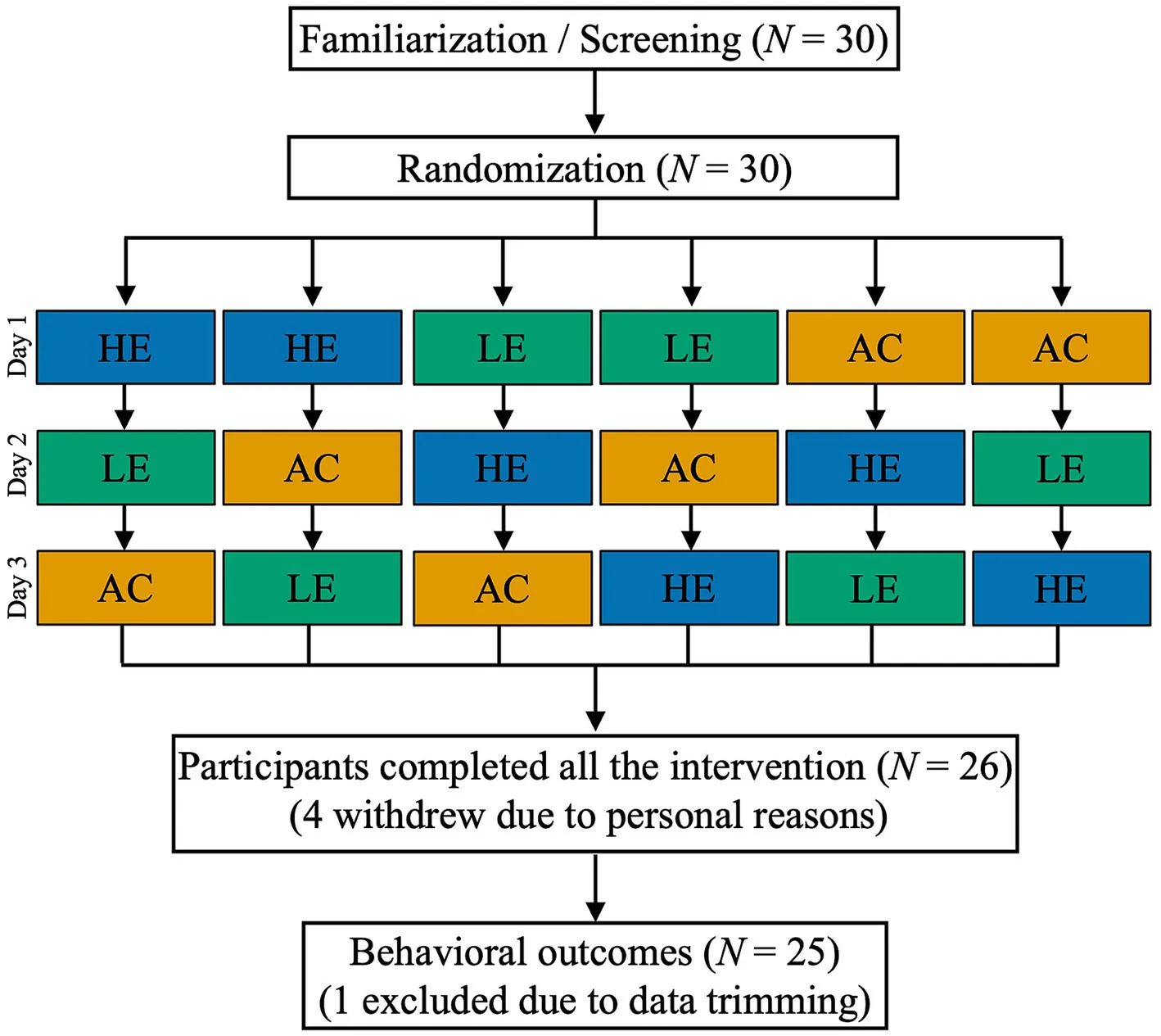
Diagram of participants’ enrollment. HE, high cognitive demanding exercise; LE, low cognitively demanding exercise; AC, active control.

To ensure consistency in participants’ baseline states, all individuals completed a familiarization phase prior to the intervention sessions. Following this phase, the first intervention was scheduled no sooner than 72 h later to allow adequate recovery and to minimize potential confounding effects. Additionally, a minimum 72-h interval was maintained between each of the three intervention sessions to reduce the risk of carryover effects. To minimize potential circadian variation, all three experimental sessions were administered at approximately the same time of day for each participant (Van Dongen et al., 2016). Before each intervention, participants completed the Felt Arousal Scale (FAS) and the Feeling Scale (FS) to confirm that their arousal and mood levels remained stable across all conditions, thereby ensuring comparability of psychological states prior to each session; the inhibitory control task was delivered before and after (10 min) each session. The heart rate was recorded throughout the intervention in the subsample of participants. Immediately after the intervention, all participants completed the OMNI ratings of perceived exertion (RPE) and the mental effort rating scale (MERS). The entire procedure and informed consent were approved by the Institutional Review Board of National Taiwan Normal University.

### 2.4 Intervention

A 20-min interval training was implemented in the exercise condition (i.e., HE and LE), which includes 2 min of exercise and 30 s of rest for a total of 8 rounds. These interval types of exercise were prescribed to better mimic children’s physical activity patterns during unstructured recess (Ridgers et al., 2012; Ridgers et al., 2009). Moreover, previous literature found that children outperformed in the Stroop task in terms of reaction time following intermittent exercise compared with continuous forms of aerobic exercise (Lambrick et al., 2016).

The cognitive demands were manipulated using the BlazePod light system (Play Coyotta Ltd., Aviv, Israel). Three light sensors were mounted 1 meter high on a white wall, forming a triangle with sides of 50 cm. Participants began each exercise bout by running in place at a starting line positioned 2 meters from the wall. When a light stimulus is activated, the participant is required to run forward to tap the designated sensor and then return to the starting line before responding to the next stimulus. Each sensor remains illuminated for a maximum duration of 2 s and is automatically turned off if not tapped within this time period. The light stimulus was presented at random intervals of 1–3 s.

The levels of cognitive demands in the HE and LE were adapted from Chueh et al. (2023). In this study, HE and LE referred to exercise conditions with relatively higher and lower cognitive demands, respectively. These labels described the relative differences between the two conditions rather than the absolute levels of task difficulty.

In both exercise conditions, the participants were instructed to tap the red target as quickly and accurately as possible. In the LE condition, one of the three sensors was illuminated in red at a randomly selected position. In the HE condition, all three sensors lit up simultaneously at random positions, with one red target and two differently colored distractors. This manipulation was designed to conceptually resemble the contrast in interference-control demands between congruent (lower interference demand) and incongruent (higher interference demand) Flanker trials, rather than to reproduce the same cognitive processes. Specifically, the HE additionally required participants to identify the target and filter out the distractors. These additional cognitive processes were expected to place greater demands on interference control in HE than in LE.

In the AC condition, participants completed two rounds of 10 stretching exercises, with each stretch held for a duration of 30 s. An active rather than passive control condition was selected because the intervention was conducted during scheduled physical education classes. This ensured that participants were not deprived of opportunities for physical activity during regular class time.

### 2.5 Reaction-time preprocessing

#### 2.5.1 Data trimming

The trial-level reaction times were preprocessed prior to aggregation. Trials with implausible latencies (≤0 ms) or anticipatory responses (<200 ms) were excluded (Whelan, 2008). Omission trials and responses exceeding the 1,000-ms response window were coded as incorrect; they were retained for accuracy computations but excluded from RT averaging. Given the positively skewed nature of reaction time distribution (Ratcliff, 1993), outliers were trimmed at the Individual × Condition (HE, LE, AC) × Task (congruent, incongruent) level using the median absolute deviation (MAD) method (Leys et al., 2013). Specifically, trials with RTs falling outside the range of median ± 3 × MAD were excluded.

After MAD-based trimming, the average proportion of the excluded trials across participants was 9.3 and 9.9% in the pre- and post-test congruent trials, and 12.3 and 13.0% in the pre- and post-test incongruent trials. Notably, one participant contributed no valid trials in the LE-incongruent condition at post-test due to the absence of correct responses. Therefore, this cell was excluded from subsequent analyses of incongruent reaction time, incongruent accuracy, and the Flanker effect.

#### 2.5.2 Exploratory distributional analyses

For the exploratory distributional analyses, all valid trials (correct and error responses) in the post-test for each condition were included for the analysis of CAF and DP. The trials were vincentized into five equipopulated RT bins (fast to slow) for each participant and task (Hübner and Töbel, 2019; Ponce et al., 2025). Omission errors were excluded from the computation (Heitz and Engle, 2007; Kao et al., 2017a).

To capture the dynamic conflict monitoring, the mean accuracy for each bin was plotted against the reaction distribution for congruent and incongruent trials. As CAF effects are theoretically expected to emerge primarily under incongruent trials (Heitz, 2014; Ridderinkhof, 2002), incongruent trials were analyzed separately to account for differences in conflict demands. To visualize the differences in the dynamics of conflict processing under each condition, the Flanker effect was calculated for each bin as the difference between the incongruent and congruent mean reaction times for each participant. The resulting dataset therefore contained three conditions (HE, LE, and AC) × five bins for CAF analysis, and three conditions × four segments for DP analysis, representing the magnitude of interference across the reaction time distribution.

### 2.6 Statistical analysis

Statistical analyses were performed using JASP (version 0.95.1; JASP Team, 2025). The significance level was set at α = 0.05. All tables were generated using Microsoft Excel and manually annotated for clarity. The visualization of CAF and DP were generated in R statistical software (v4.5.1; R Core Team, 2025) using the following packages: *readr* (v2.1.6; Wickham et al., 2025a, for data import), *stringr* (v1.6.0; Wickham, 2025, for file parsing), *dplyr* (v1.1.4; Wickham et al., 2023, for data binning), *tidyr* (v.1.3.2; Wickham et al., 2025b, for data reshaping in DP), and *ggplot 2* (Wickham, 2016, for plotting CAF and DP).

#### 2.6.1 Demographic and manipulation check

Descriptive statistics (means and standard deviations) were used to summarize the demographic variables (age, BMI) and cognitive performance. To examine the manipulation of cognitive demand and exercise intensity, separate one-way repeated-measures ANOVAs were conducted on MERS, heart rate, and OMNI scores across HE, LE, and AC. In addition, a one-way repeated-measures ANOVA was conducted to assess the differences in affective responses (FS, FAS) at baseline across conditions.

#### 2.6.2 Primary analyses

The primary analyses were to evaluate the effects of cognitive demand on Flanker task performance. To control for baseline differences across participants and to increase the precision of estimated condition effects by minimizing day-to-day variability, change scores were computed for each participant and each outcome measure by subtracting pre-intervention values from post-intervention values (Luck and Gaspelin, 2017).

A 3 (Condition) × 2 (Congruency) repeated-measures ANOVA was conducted on reaction time (H1) and accuracy (H2). In addition, a one-way repeated-measures ANOVA was conducted to investigate the differences in Flanker effect (H1) across conditions.

Greenhouse–Geisser corrections were applied where the assumption of sphericity was violated. Where significant interactions were found, pairwise comparisons with Bonferroni correction were performed to further explore differences across conditions and congruency.

#### 2.6.3 Exploratory secondary analyses

Following completion of data collection and a subsequent review of the relevant literature, CAF and DP analyses were added as exploratory secondary analyses. Two repeated-measures ANOVAs were conducted for the CAF and DP analyses, respectively.

CAF data were analyzed to examine whether exercise effects selectively emerged across different bins of the reaction time distribution. Repeated-measures ANOVAs with Condition (HE, LE, and AC) and Bin (1–5) as within-subject factors, separately for congruent and incongruent trials, were conducted.

To evaluate the temporal dynamics of the interference effect (i.e., Flanker effect) (Ridderinkhof, 2002), delta plots were computed for each participant and condition by calculating the mean reaction time differences between incongruent and congruent trials across five bins. Segment-wise slopes were derived between consecutive bins (e.g., Slope_1-2_ = FE_2_ – FE_1_) to represent how interference changed across consecutive reaction time distributions. Negative slopes indicate that the interference effect decreased for slower responses, reflecting more effective suppression, whereas positive slopes suggest an increase in interference with slower responses.

A 3 (Condition: HE, LE, and AC) × 4 (Segment: Slope_1-2_, Slope_2-3_, Slope_3-4_, Slope_4-5_) repeated measures ANOVA was conducted to test whether the temporal dynamics of interference suppression differed across conditions (Ridderinkhof, 2002; van den Wildenberg et al., 2010).

Greenhouse–Geisser corrections were applied where the assumption of sphericity was violated. Where significant interactions were found, pairwise comparisons with Bonferroni correction were performed to further explore differences across conditions.

## 3 Results

### 3.1 Participants characteristics

Table 1 presented the characteristics of the participants in the present study.

**Table 1:** Demographics of the participants.

Measure	M (SD)
*N*	26
Age (year)	11.8 (0.5)
Height (cm)	153.9 (5.6)
Weight (kg)	40.1 (7.1)
BMI (kg/m^2^)	16.8 (2.7)

*N*, sample size; BMI, body mass index; SD, standard deviation.

### 3.2 Baseline check for affective measures

There were no significant baseline differences in affective responses across conditions, as indicated by the non-significant main effects of condition for the Felt Arousal Scale (FAS) and the Feeling Scale (FS) (see Table 2).

**Table 2:** Descriptive and inferential statistics for affective measures and manipulation checks across conditions.

Measure (*N*)	HE, M (SD)	LE, M (SD)	AC, M (SD)	F (df1, df2)	*p*	$\eta_{p}^{2}$	Pairwise comparisons
Pre-test							
FAS (26)	4.3 (0.9)	4.6 (1.3)	4.2 (1.2)	1.77 (1.58, 39.46)	0.189	0.07	—
FS (26)	3.3 (1.5)	3.1 (2.3)	3.6 (1.7)	1.18 (1.54, 38.48)	0.306	0.05	—
During							
HR, bpm (14)	166.3 (10.3)	165.7 (11.7)	113.4 (11.3)	99.16 (2, 26)	<0.001	0.88	HE > AC: *t* (13) = 12.71, *p* < 0.001, *d* = 4.77, 95% CI [1.95, 7.48] LE > AC: *t* (13) = 11.80, *p* < 0.001, *d* = 4.71, 95% CI [2.00, 7.54] HE vs. LE: *t* (13) = 0.15, *p* = 1.00, *d* = 0.06, 95% CI [−1.01, 1.13]
Post-test							
OMNI RPE (26)	7.3 (2.2)	7.2 (2.1)	2.0 (1.0)	131.67 (1.31, 32.78)	<0.001	0.84	HE > AC: *t* (25) = 11.62, *p* < 0.001, *d* = 2.85, 95% CI [1.64, 4.07] LE > AC: *t* (25) = 12.62, *p* < 0.001, *d* = 2.79, 95% CI [1.63, 3.95] HE vs. LE: *t* (25) = 0.57, *p* = 1.00, *d* = 0.06, 95% CI [−0.22, 0.34]
MERS (26)	4.0 (1.7)	2.8 (1.8)	1.7 (0.8)	26.72 (2.00, 49.85)	<0.001	0.52	HE > LE: *t* (25) = 3.76, *p* = 0.003, *d* = 0.77, 95% CI [0.17, 1.36] HE > AC: *t* (25) = 7.16, *p* < 0.001, *d* = 1.54, 95% CI [0.75, 2.32] LE > AC: *t* (25) = 3.64, *p* = 0.004, *d* = 0.77 95% CI [0.16, 1.38]

*N*, sample size; HE, high cognitively demanding exercise; LE, low cognitively demanding exercise; AC, active control; FAS, Felt Arousal Scale; FS, Feeling Scale; MERS, Mental Effort Rating Scale; HR, heart rate (beats per minute); OMNI RPE, child OMNI Rating of Perceived Exertion.

### 3.3 Manipulation checks for cognitive engagement during exercise

The main effect of conditions in MERS scores indicated a successful manipulation of cognitive demand, *F* (2, 49.85) = 26.72, *p* < 0.001, $\eta_{p}^{2}$ = 0.52.

The HE elicited significantly greater perceived cognitive demand than both LE and AC. In addition, LE showed greater perceived cognitive demand than AC (see Table 2; Figure 2).

**Figure 2:**
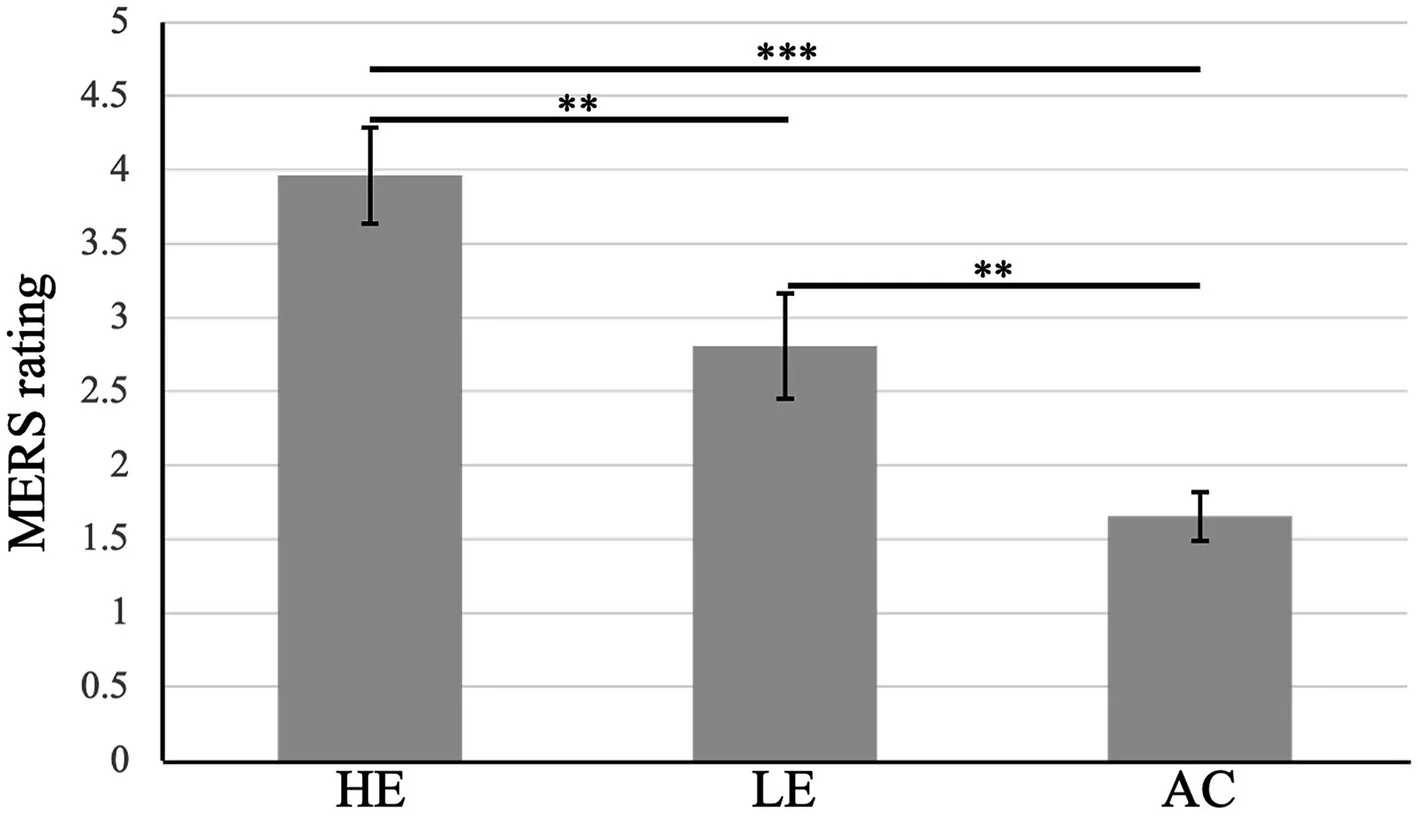
Comparisons of MERS among HE, LE, and AC. HE, high cognitive demanding exercise; LE, low cognitively demanding exercise; AC, active control; MERS, mental effort rating scale (error bars show standard errors). ***p < 0.001; **p < 0.01.

### 3.4 Manipulation checks for exercise intensity

A significant main effect of conditions was observed for both heart rate (HR), *F* (2, 26) = 99.16, *p* < 0.001, $\eta_{p}^{2}$ = 0.88, and OMNI RPE scale, *F* (1.31, 32.78) = 131.67, *p* < 0.001, $\eta_{p}^{2}$ = 0.84 (see Table 2).

Post-hoc analyses revealed that both the HE and LE elicited higher HR and OMNI than AC. No significant differences were observed between the two exercise conditions for either heart rate or OMNI RPE scale (see Table 2).

### 3.5 Measures for reaction time

A significant main effect of congruency was observed, *F* (1, 24) = 34.53, *p* < 0.001, $\eta_{p}^{2}$ = 0.59, indicating that the participants exhibited a greater change on incongruent compared with congruent trials (see Table 3 Panel B).

**Table 3:** Reaction time across conditions and inferential tests.

Panel A. Descriptive statistics				
Variables (*N*)	Time	HE, M (SD)	LE, M (SD)	AC, M (SD)
Congruent RT (ms) (*N* = 26)	Pre	461.6 (54.2)	441.0 (39.7)	454.2 (58.6)
	Post	442.0 (43.9)	432.3 (53.1)	473.7 (63.7)
	ΔRT	−19.6 (36.1)	−8.7 (30.3)	19.5 (54.9)
Incongruent RT (ms) (HE = 26; LE = 25; AC = 26)	Pre	549.6 (77.1)	516.8 (53.0)	536.9 (66.6)
	Post	507.9 (50.2)	491.3 (51.5)	511.1 (73.0)
	ΔRT	−41.7 (50.3)	−21.6 (25.4)	−25.8 (58.1)
Flanker effect (ms) (HE = 26; LE = 25; AC = 26)	Pre	88.0 (40.4)	75.8 (31.8)	82.7 (28.4)
	Post	65.9 (34.4)	58.5 (39.4)	37.4 (47.5)
	ΔRT	−22.1 (27.9)	−12.9 (29.3)	−45.3 (47.1)
One participant was excluded from the LE incongruent RT cell after trimming because no valid responses remained. RT, reaction time; ΔRT, post-test minus pre-test; HE, high cognitively demanding exercise; LE, low cognitively demanding exercise; AC, active control.				

Panel B. Inferential statistics					
Variables (*N*)	Effect	F (df1, df2)	*p*	$\eta_{p}^{2}$	Pairwise comparisons
RT change score (*N* = 25)	Condition	2.53 (1.35, 32.41)	0.112	0.10	—
	Congruency	34.53 (1, 24)	<0.001	0.59	Incongruent > congruent: *t* (24) = 5.88, *p* < 0.001, *d* = 0.60, 95% CI [0.32, 0.87]
	Condition × Congruency	4.93 (1.67, 40.13)	0.017	0.17	No condition differences within congruent or incongruent (all *p* > 0.05)
Flanker-effect change score (*N* = 25)	Condition	4.93 (1.67, 40.13)	0.017	0.17	AC showed a greater reduction than LE: *t* (24) = −2.73, *p* = 0.035, *d* = −0.81, 95% CI [−1.64, 0.01] HE vs. LE: *t* (24) = 1.04, *p* = 0.925, *d* = 0.21, 95% CI [−0.31, 0.73] HE vs. AC: *t* (24) = −2.03, *p* = 0.160, *d* = −0.61, 95% CI [−1.40, 0.19]

One participant was excluded from the LE incongruent RT cell after trimming because no valid responses remained. RT, reaction time; HE, high cognitively demanding exercise; LE, low cognitively demanding exercise; AC, active control.

In addition, the Condition × Congruency interaction was significant, *F* (1.67, 40.13) = 4.93, *p* = 0.017, $\eta_{p}^{2}$ = 0.17. Post-hoc pairwise comparisons indicated that there were no significant differences in change scores across conditions for either congruent trials (all *p* > 0.05) or incongruent trials (all *p* > 0.05) (see Table 3 Panel B).

In terms of the Flanker effect, a significant main effect of condition was observed, *F* (1.67, 40.13) = 4.93, *p* = 0.017, $\eta_{p}^{2}$ = 0.17. *Post hoc* pairwise comparisons indicated that the LE showed a significantly smaller reduction in Flanker effect compared with AC (see Table 3 Panel B). No significant differences were observed between HE and LE, and HE and AC (see Table 3 Panel B).

### 3.6 Measures for accuracy

The results indicated that there was no significant main effect of Condition, *F* (1.34, 32.13) = 0.81, *p* = 0.41, $\eta_{p}^{2}$ = 0.03, nor the Condition × Congruency interaction, *F* (1.15, 27.49) = 1.32, *p* = 0.27, $\eta_{p}^{2}$ = 0.05. However, a significant main effect of Congruency, *F* (1, 24) = 4.83, *p* = 0.04, $\eta_{p}^{2}$ = 0.17, was observed, indicating that the participants showed greater change in accuracy on incongruent trials compared to congruent trials (see Table 4 Panel B).

**Table 4:** Accuracy across conditions and inferential tests.

Panel A. Descriptive statistics				
Variables (*N*)	Time	HE, M (SD)	LE, M (SD)	AC, M (SD)
Congruent ACC (*N* = 26)	Pre	0.971 (0.100)	0.964 (0.100)	0.918 (0.200)
	Post	0.972 (0.100)	0.972 (0.090)	0.966 (0.100)
	ΔACC	0.001 (0.020)	0.008 (0.042)	0.048 (0.151)
Incongruent ACC (HE = 26; LE = 25; AC = 26)	Pre	0.928 (0.100)	0.900 (0.200)	0.931 (0.100)
	Post	0.929 (0.100)	0.904 (0.200)	0.929 (0.100)
	ΔACC	0.001 (0.083)	0.004 (0.076)	−0.002 (0.140)
One participant was excluded from the LE incongruent ACC cell after trimming because no valid responses remained. Accuracy is expressed as a proportion ranging from 0 to 1. ACC, accuracy; ΔACC, post-test minus pre-test; HE, high cognitively demanding exercise; LE, low cognitively demanding exercise; AC, active control.				

Panel B. Inferential statistics					
Analysis (*N*)	Effect	F (df1, df2)	*p*	$\eta_{p}^{2}$	Pairwise comparisons
ACC change score (*N* = 25)	Condition	0.81 (1.34, 32.13)	0.409	0.03	—
	Congruency	4.83 (1, 24)	0.038	0.17	Incongruent < Congruent: *t* (24) = 2.20, *p* = 0.038, *d* = 0.24, 95% CI [0.01, 0.46]
	Condition × Congruency	1.32 (1.15, 27.49)	0.267	0.05	—

One participant was excluded from the LE incongruent ACC cell after trimming because no valid responses remained. ACC, accuracy; HE, high cognitively demanding exercise; LE, low cognitively demanding exercise; AC, active control.

### 3.7 Exploratory analysis of the conditional accuracy function (CAF)

To examine condition effects on accuracy across the reaction time distribution, repeated-measures ANOVA were conducted with Condition (HE, LE, and AC) and Bin (1–5) as within-subject factors, separately for congruent and incongruent trials (see Table 5).

**Table 5:** Conditional accuracy function by bin and condition.

Panel A. Congruent trials			
Variable	HE, M (SD) (*N* = 26)	LE, M (SD) (*N* = 26)	AC, M (SD) (*N* = 26)
Bin 1			
Accuracy	0.961 (0.100)	0.972 (0.100)	0.948 (0.200)
Reaction time	350.8 (29.6)	346.7 (28.1)	368.5 (38.3)
Bin 2			
Accuracy	0.978 (0.100)	0.968 (0.100)	0.961 (0.100)
Reaction time	399.4 (37.4)	390.5 (37.9)	418.0 (49.5)
Bin 3			
Accuracy	0.982 (0.100)	0.977 (0.100)	0.969 (0.100)
Reaction time	435.2 (44.1)	425.9 (50.3)	458.4 (62.0)
Bin 4			
Accuracy	0.970 (0.100)	0.978 (0.100)	0.972 (0.100)
Reaction time	478.8 (53.2)	467.1 (68.8)	507.9 (78.7)
Bin 5			
Accuracy	0.967 (0.100)	0.963 (0.100)	0.962 (0.100)
Reaction time	549.7 (69.3)	535.0 (90.3)	585.5 (104.2)

Panel B. Incongruent trials			
Variable (*N*)	HE, M (SD) (*N* = 26)	LE, M (SD) (*N* = 25)	AC, M (SD) (*N* = 26)
Bin 1			
Accuracy	0.835 (0.200)	0.849 (0.200)	0.881 (0.200)
Reaction time	410.7 (40.2)	404.3 (37.8)	423.1 (57.8)
Bin 2			
Accuracy	0.945 (0.100)	0.967 (0.100)	0.941 (0.100)
Reaction time	466.3 (42.9)	455.0 (43.6)	474.8 (61.4)
Bin 3			
Accuracy	0.972 (0.100)	0.959 (0.100)	0.926 (0.100)
Reaction time	502.3 (49.5)	487.8 (51.2)	514.8 (69.6)
Bin 4			
Accuracy	0.941 (0.100)	0.961 (0.100)	0.940 (0.100)
Reaction time	548.2 (61.7)	527.1 (63.6)	562.7 (76.7)
Bin 5			
Accuracy	0.956 (0.100)	0.961 (0.100)	0.944 (0.100)
Reaction time	618.1 (77.7)	589.8 (84.4)	635.1 (90.9)

Panel C. Inferential statistics					
Trial type (*N*)	Effect	F (df1, df2)	*p*	$\eta_{p}^{2}$	Pairwise comparisons
Congruent (*N* = 26)	Condition	1.45 (1.53, 38.12)	0.250	0.06	—
	Bin	1.74 (1.38, 34.59)	0.200	0.07	—
	Condition × Bin	1.45 (2.09, 53.35)	0.480	0.03	—
Incongruent (*N* = 25)	Condition	1.07 (1.92, 46.07)	0.350	0.04	—
	Bin	11.33 (1.29, 30.89)	<0.001	0.32	Bin 1 < Bin 2: *t* (24) = −4.34, *p* = 0.002, *d* = −0.77, 95% CI [−1.41, −0.12] Bin 1 < Bin 3: *t* (24) = −3.72, *p* = 0.011, *d* = −0.80, 95% CI [−1.56, −0.05] Bin 1 < Bin 4: *t* (24) = −3.15, *p* = 0.043, *d* = −0.72, 95% CI [−1.50, 0.06] Bin 1 < Bin 5: *t* (24) = −3.44, *p* = 0.021, *d* = −0.78, 95% CI [−1.56, 0.002]
	Condition × Bin	1.31 (2.82, 67.68)	0.280	0.05	—

One participant was excluded from the LE incongruent RT and ACC cell after trimming because no valid responses remained. Accuracy is expressed as a proportion ranging from 0 to 1, and reaction time is expressed in milliseconds. HE, high cognitively demanding exercise; LE, low cognitively demanding exercise; AC, active control.

For accuracy in congruent trials, there were no significant main effects of Condition, *F* (1.53, 38.12) = 1.45, *p* = 0.25, $\eta_{p}^{2}$ = 0.06, or Bin, *F* (1.38, 34.59) = 1.74, *p* = 0.20, $\eta_{p}^{2}\;$ = 0.07, and the Condition × Bin interaction was also not significant, *F* (2.09, 53.35) = 1.45, *p* = 0.48, $\eta_{p}^{2}$ = 0.03 (see Table 5 Panel C).

For accuracy in incongruent trials, there was no significant main effect of Condition, *F* (1.92, 46.07) = 1.07, *p* = 0.35, $\eta_{p}^{2}$ = 0.04, and no Condition × Bin interaction, *F* (2.82, 67.68) = 1.31, *p* = 0.28, $\eta_{p}^{2}$ = 0.05. However, a significant main effect of Bin, *F* (1.29, 30.89) = 11.33, *p* < 0.001, $\eta_{p}^{2}$ = 0.32, was found, indicating increased accuracy across slower reaction time bins. Post-hoc test for the simple effect of Bin suggested that the first bin was significantly less accurate than the rest of the bins (see Table 5 Panel C).

### 3.8 Exploratory analysis of the delta-plot (DP)

To examine the interference effects among conditions across the reaction time distribution, repeated-measures ANOVA was conducted with Condition (HE, LE, AC) and Segment (Slope_1-2_, Slope_2-3_, Slope_3-4_, Slope_4-5_) on the slope values as within-subject factors (Figure 3).

**Figure 3:**
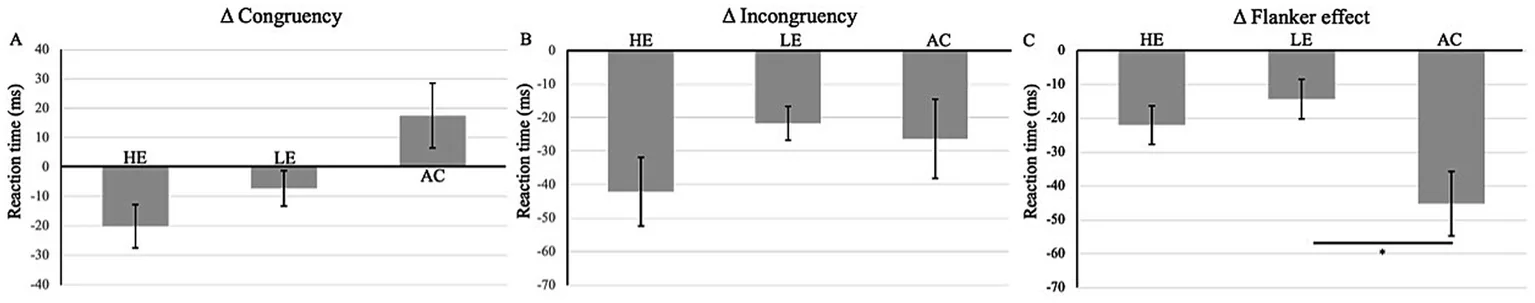
Comparison of reaction time among HE, LE, and AC. Three panels present the change scores across conditions. **(A)** congruent trials. **(B)** Incongruent trials. **(C)** Flanker effect. HE, high cognitive demanding exercise; LE, low cognitively demanding exercise; AC, active control; error bars show standard errors. *p < 0.05.

The results suggested no significant difference in the main effect of condition, *F* (1.57, 37.75) = 0.26, *p* = 0.72, $\eta_{p}^{2}$ = 0.01, nor the interaction of Condition × Segment, *F* (3.02 72.58) = 0.53, *p* = 0.67, $\eta_{p}^{2}$ = 0.02. However, a significant main effect of Segment, *F* (2.56, 61.47) = 7.26, *p* < 0.001, $\eta_{p}^{2}$ = 0.23, was found (see Table 6 Panel B). Post-hoc comparisons showed that the slope between the first and second segment was significantly larger than those between the later segments (see Table 6 Panel B).

**Table 6:** Delta-plot slopes by segment and condition.

Panel A. Descriptive statistics			
Variable	HE, M (SD) (*N* = 25)	LE, M (SD) (*N* = 25)	AC, M (SD) (*N* = 25)
Segment 1 (Slope_1–2_)	6.99 (13.43)	6.93 (14.21)	2.38 (15.16)
Segment 2 (Slope_2–3_)	0.03 (13.10)	−2.58 (13.76)	0.12 (14.49)
Segment 3 (Slope_3–4_)	0.99 (17.90)	−2.24 (15.48)	−0.30 (14.50)
Segment 4 (Slope_4–5_)	−1.61 (19.39)	−5.42 (17.20)	−4.09 (27.73)
One participant was excluded from the LE incongruent RT cell after trimming because no valid responses remained. Values are means (standard deviations). Slopes are expressed in milliseconds and represent changes in the Flanker effect between consecutive reaction time bins. HE, high cognitively demanding exercise; LE, low cognitively demanding exercise; AC, active control.			

Panel B. Inferential statistics				
Effect (*N*)	F (df1, df2)	*p*	$\eta_{p}^{2}$	Pairwise comparisons
Condition (*N* = 25)	0.26 (1.57, 37.75)	0.718	0.01	—
Segment (*N* = 25)	7.26 (2.56, 61.47)	< 0.001	0.23	Segment 1 > Segment 2: *t* (24) = 3.11, *p* = 0.029, *d* = 0.37, 95% CI [0.01, 0.73] Segment 1 > Segment 3: *t* (24) = 2.97, *p* = 0.040, *d* = 0.35, 95% CI [−0.01, 0.72] Segment 1 > Segment 4: *t* (24) = 3.77, *p* = 0.006, *d* = 0.54, 95% CI [0.13, 0.96]
Condition × Segment (N = 25)	0.53 (3.02, 72.58)	0.667	0.02	—

One participant was excluded from the LE incongruent RT cell after trimming because no valid responses remained.

Taken together, the delta plot analysis revealed a pattern of interference scores across the reaction time distribution, consistent with the activation–suppression model. The steep positive slope in the early segment suggested that the automatic response activation dominated the fastest responses, whereas the flattening of the slope in later segments indicates the gradual engagement of top-down inhibitory control. No statistically significant exercise-condition differences were detected. Given the exploratory nature of this analysis and the limited sample size, this finding should not be interpreted as evidence that the exercise manipulation had no effect on the time course of interference suppression.

## 4 Discussion

This study examined the effects of exercise with different levels of cognitive demand on Flanker task performance among children. Under the high physical intensity elicited by the present protocol, exercise involving higher cognitive demand (HE) did not induce greater benefits in improving inhibitory control performance than exercise with lower cognitive demand (LE). However, the active control condition (AC) showed a significantly greater reduction in the Flanker effect than LE. The exploratory secondary analyses detected no statistically significant between-condition differences in post-test conditional accuracy function (CAF) or delta plot (DP) parameters.

The first hypothesis was not supported, as HE did not produce greater improvements in inhibitory-control performance than LE. This finding was inconsistent with the prediction derived from the cognitive stimulation hypothesis (Best, 2010; Pesce, 2012). Comparisons involving AC should be interpreted cautiously because AC differed from HE and LE in both physical intensity and cognitive demand, making it difficult to isolate the specific contribution of either factor.

A key methodological consideration in interpreting the lack of differential effects between HE and LE is the exercise intensity achieved during the intervention. Although the protocol targeted a moderate-to-vigorous intensity range, heart rate data from the monitored subsample revealed that both exercise conditions exceeded the prespecified range (HE: 82.7% HR_max_; LE: 83.0% HR_max_). This unexpected physical intensity is critical, as higher exercise loads may negatively affect attentional processes (Schmidt et al., 2015) or fail to confer additional benefits for inhibitory control (Jäger et al., 2015) in children. Accordingly, the combination of the elevated physical load and the additional cognitive demand of HE may have increased overall task load, thereby attenuating or masking the potential benefits of cognitive engagement (Gallotta et al., 2012, 2015). This deviation from the prescribed intensity therefore limits conclusions about whether different levels of cognitive demand would produce differential effects when exercise is performed within the intended intensity range (Anzeneder et al., 2023b). This finding underscores the importance of carefully controlling exercise intensity when attempting to isolate the contribution of cognitive demand (Figure 4).

**Figure 4:**
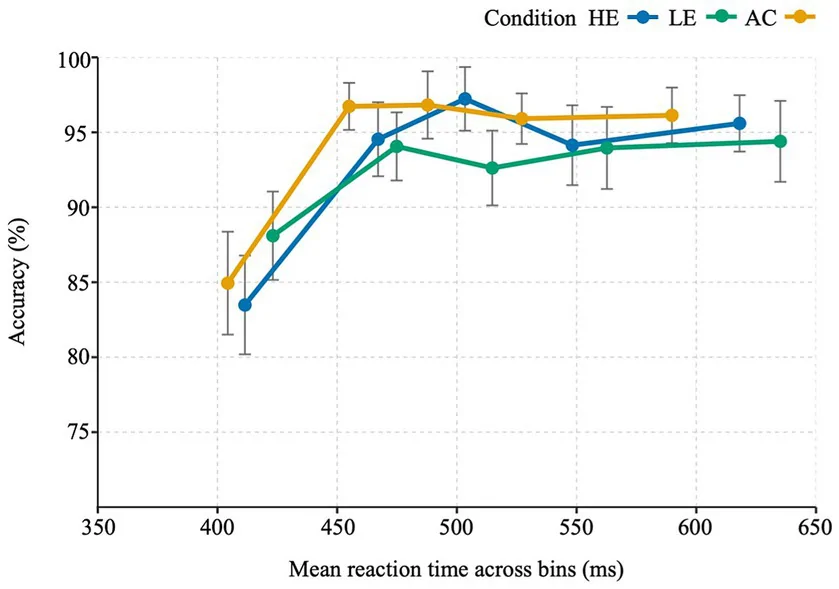
Conditional accuracy function in incongruent trials among three conditions. HE, high cognitive demanding exercise; LE, low cognitively demanding exercise; AC, active control; (error bars show standard errors).

The significantly greater reduction in the Flanker effect following AC than LE was also contrary to our expectations. Additional decomposition analyses showed that incongruent reaction time decreased from pre- to post-test across all three conditions, with no significant differences in the magnitude of change among conditions (see Supplementary material, Decomposition of Flanker effect). In contrast, congruent reaction time decreased in HE and LE but increased by 19.5 ms in AC. Thus, the comparatively large reduction in the Flanker effect following AC may not reflect a selective improvement in inhibitory control performance but may instead have been driven by the different directions of change in congruent and incongruent reaction times.

The use of stretching as an active control may have reduced the sensitivity to detect condition-specific effects. This condition was selected to avoid withholding opportunities for physical activity because the intervention was conducted during scheduled physical education classes. Previous research has suggested that the beneficial effects of acute cognitively demanding exercise may be more evident when compared with an inactive control (Cabral et al., 2026). Stretching has been shown to improve Stroop task performance relative to seated rest, potentially through an increase in subjective vigor (Sudo and Ando, 2020). The potential cognitive and affective effects of stretching may therefore have attenuated the contrast between conditions.

Taken together, the findings did not demonstrate an additional benefit of higher cognitive demand for inhibitory control under high physiological load in the present protocol. Nevertheless, a greater improvement in incongruent trials compared with congruent trials across conditions suggested that acute exercise may enhance executive processes involved in conflict resolution and response inhibition (Álvarez-Bueno et al., 2017; Oberste et al., 2019; Verburgh et al., 2014). Future studies should further disentangle the effects of exercise intensity and cognitive demand to clarify their combined influence on inhibitory control outcomes.

The second hypothesis regarding accuracy was not supported. Accuracy did not differ significantly across conditions, replicating the null findings frequently reported in acute exercise studies employing inhibitory control tasks (Anzeneder et al., 2023a, 2023b; Bedard et al., 2021; Bulten et al., 2022; Ludyga et al., 2024).

The absence of statistically significant condition-related differences in accuracy may partly reflect the characteristics of the present sample. Although acute-exercise effects on accuracy may not differ across fitness levels (Ludyga et al., 2016; Oberste et al., 2019), children with higher aerobic fitness have exhibited less interference than their low-fit counterparts (Raine et al., 2018) and shown more adult-like Flanker task performance (Kao et al., 2017b) in the Flanker task, which may reduce the sensitivity of accuracy measures to detect acute exercise-induced changes. In this context, a more complex cognitive task may be required to detect exercise-induced differences in accuracy (McMorris and Hale, 2012).

Additional analyses suggested that neither congruent nor incongruent accuracy showed a significant Condition × Time interaction, indicating that changes in accuracy did not differ significantly across conditions. Nevertheless, the descriptive results in AC revealed a pattern consistent with a possible speed-accuracy adjustment. Specifically, congruent reaction time increased by 19.5 ms from pre- to post-test, accompanied by a 4.8-percentage-point increase in congruent accuracy. Although this pattern may reflect a shift toward a slower and more accurate response strategy, it should be interpreted cautiously because the Condition × Time interaction for congruent accuracy was not significant. Further research is needed to clarify the mechanisms underlying this condition-specific response pattern (Figure 5).

**Figure 5:**
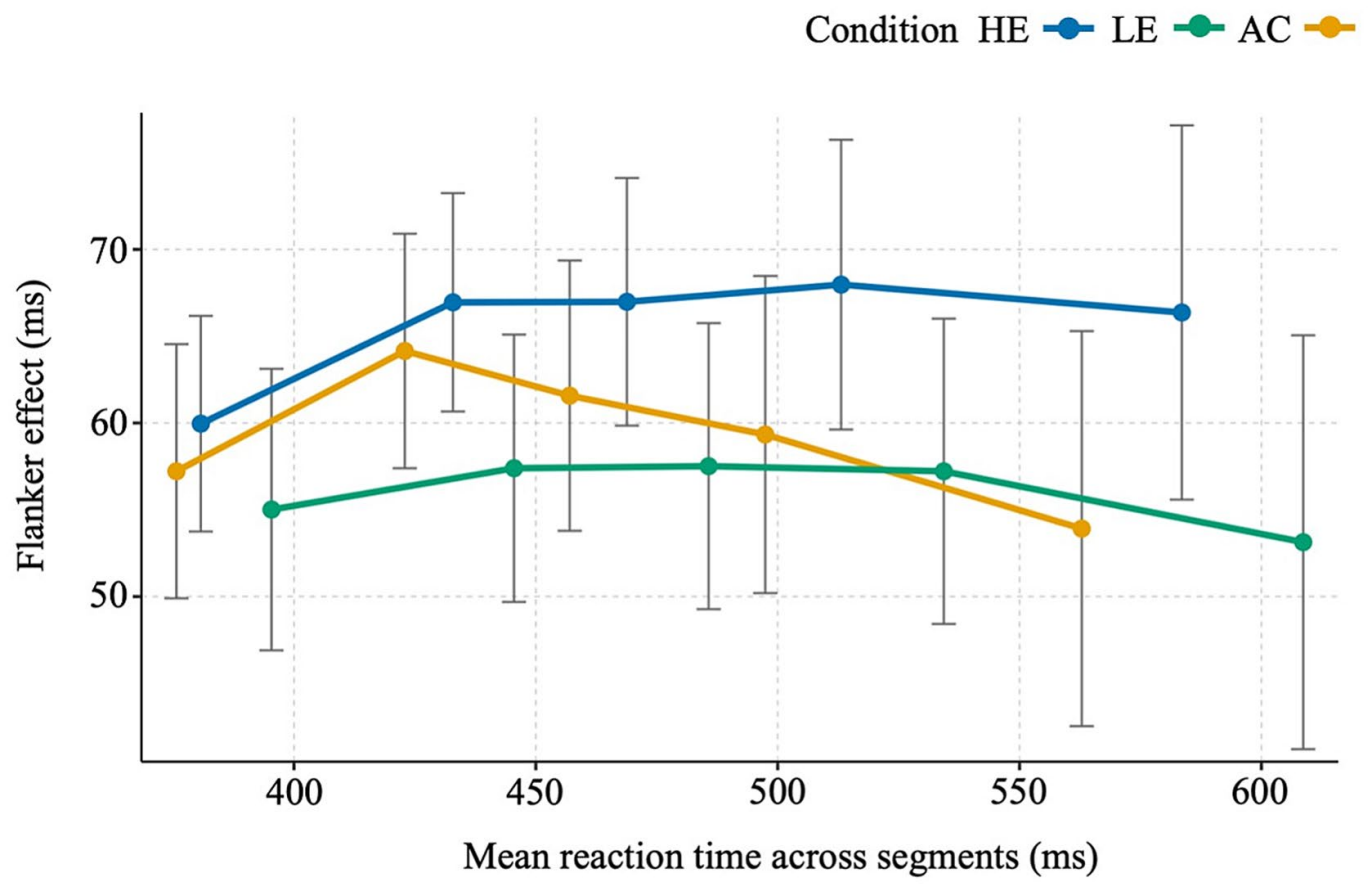
Delta plot among three conditions. HE, high cognitive demanding exercise; LE, low cognitively demanding exercise; AC, active control; RT, reaction time (error bars show standard errors).

As exploratory secondary analyses, CAF and DP were used to examine whether performance patterns varied across the reaction-time distribution among conditions. The CAF analysis examined patterns in congruent and incongruent trials, whereas the DP analysis examined segment-specific patterns of the interference effect.

No statistically significant condition-related interaction effects were detected in the exploratory distributional analyses. Previous studies have similarly suggested that acute exercise may not alter the shape of the inhibition process across reaction time distribution (Davranche et al., 2006) but may facilitate response execution through reductions in premotor time, as indicated by earlier EMG onset (Davranche et al., 2005). However, it is noteworthy that the present sample size was determined based on the primary reaction time outcome; the study may have had limited sensitivity to detect subtle condition-related interactions in these distributional patterns.

Subtle modulations in cognitive control following cognitively demanding exercise may therefore be more readily detectable using neural measures than through behavioral distributional indices alone. For instance, Chueh et al. (2023) reported earlier peaks of conflict-related ERPs (e.g., N2) following high cognitively demanding exercise compared with low-demanding exercise in young adults, suggesting more efficient cortical conflict evaluation. In contrast, Ludyga et al. (2024) found favorable hemodynamic alterations in the prefrontal cortex of children following exercise with low cognitive demand, particularly during an emotional conflict task. Taken together, future studies would benefit from incorporating neural and kinematic assessments to more comprehensively characterize exercise-induced changes in cortical processing and motor execution underlying cognitive enhancements.

Within these exploratory analyses, the main effects observed in the CAF and DP revealed canonical patterns of inhibitory control. Specifically, the CAF demonstrated a typical speed–accuracy profile, with higher error rates among the fastest responses that declined as reaction time increased (Ridderinkhof, 2002). Similarly, the DP analysis showed a steep positive slope in the earliest segments, followed by flatter slopes in later segments. These patterns reflect the gradual engagement of top-down inhibitory control described by the activation-suppression model, in which early responses are dominated by automatic activation, whereas later responses reflect increasing suppression of interference (Ridderinkhof, 2002; Van Den Wildenberg et al., 2010).

These inconsistencies across studies may also be interpreted within the strength model of self-control (Baumeister et al., 1998, 2007). Children may have exerted greater effort during cognitively demanding exercise, thereby reducing the resources available for subsequent executive control tasks (Audiffren, 2009; Audiffren and André, 2015). Developmental differences in resource regulation may further amplify such resource depletion. Children appear to be more susceptible to self-control depletion during motor tasks than young adults (Strobach and Karbach, 2024) and may prioritize motor demands at the expense of performance on concurrent cognitive tasks (Schaefer, 2014; Schaefer et al., 2008). In contrast, young adults may regulate cortical activity in a more energy-conserving manner during dual-task situations, which could help preserve the self-control resources required for subsequent cognitive tasks (de Souza et al., 2025). These developmental distinctions may partly explain why positive effects of exercise with high cognitive demand are more consistently reported in studies involving young adults (Chueh et al., 2023; Yang et al., 2025). Future studies incorporating measures of resource depletion are needed to evaluate possible developmental differences in responses to cognitively demanding exercise.

For acute interventions, both the type and magnitude of cognitive engagement may play important roles in facilitating post-exercise cognitive performance in children. For instance, Schmidt et al. (2016) found that a brief 10 min of cognitively engaging activity, rather than physical demand, improved children’s cognitive performance. Conversely, studies employing longer or more intense exercise bouts have reported less beneficial effects on attentional performance compared with aerobic exercise or cognitive training alone (Gallotta et al., 2015; Gallotta et al., 2012), and in some cases even detrimental effects on a subsequent task (Egger et al., 2018). Together, these findings suggest that excessive task complexity or prolonged cognitive load could potentially offset the benefits of acute exercise. Future studies are warranted to identify the optimal dose–response relationships for cognitively demanding exercise for promoting executive function in children.

In summary, among child athletes under the higher-than-intended physiological intensity elicited by the present protocol, no statistically significant differences in subsequent inhibitory-control performance were observed between exercise conditions involving higher and lower cognitive demand. Comparisons involving the active control should therefore be interpreted cautiously, as the observed differences may reflect variations in both physical intensity and cognitive demand. These findings underscore the need for intervention designs that carefully match physical intensity, while manipulating cognitive demand and directly assess the proposed cognitive mechanisms to clarify their contributions to post-exercise executive function performance.

### 4.1 Strength and limitations

This study has several methodological and conceptual strengths that enhance the interpretability of its findings. First, a crossover design was employed to minimize between-subject variability. Moreover, HE and LE elicited comparable levels of exercise intensity, allowing the effects of different levels of cognitive demand to be compared while physical intensity was held relatively constant (Jäger et al., 2015). All sessions were completed individually, reducing the potential influences of affective responses and social interactions (Best, 2010; Paschen et al., 2019).

Second, the present study extended the “Thinking while Moving” framework (Herold et al., 2018) to children by integrating cognitive components into the motor tasks to examine the benefits of cognitively demanding exercise. The intervention was adapted from previous research conducted with young adults (Chueh et al., 2023). MERS scores indicated greater perceived mental effort in HE than in LE, and higher scores in LE than in AC.

Third, the intervention was implemented within a naturalistic school context, with all sessions conducted either before the first class or after the regular school hours. This school-based implementation strengthens the ecological validity of the study and demonstrates that cognitively engaging exercise can feasibly be integrated into school routines.

Finally, this study included exploratory process-level analyses using CAF and DP, providing a more detailed characterization of the temporal dynamics associated with inhibitory-control performance following acute exercise. Although hypothesis-generating, these methods complemented conventional aggregated measures by describing how cognitive-control patterns unfolded across the reaction-time distribution.

Despite these strengths, several limitations of the study should be acknowledged. First, the sample consisted of typically developing school-aged children recruited from a school track-and-field team. They may therefore have had relatively high habitual physical activity levels. This specific sample may limit the generalizability of the findings to the broader population of elementary school-aged children. In addition, the present study focused exclusively on inhibitory control given its foundational role during this developmental stage. Accordingly, the findings may not extend to other domains of executive function.

Second, participants’ levels of affect and arousal were assessed only at baseline to ensure comparability across conditions. Previous studies have shown that children tend to report greater enjoyment and interest when engaging in cognitively enriched activities compared with regular exercise (Bedard et al., 2021; Kolovelonis and Goudas, 2023; Kolovelonis et al., 2023). Exercise-induced positive affect has also been suggested to contribute to improvements in executive functioning (Schmidt et al., 2016). However, because affective and arousal responses were not assessed following each intervention, the present study could not fully rule out the potential influence of condition-specific affective responses on Flanker task performance.

Third, in addition to the affective responses, relevant background variables such as physical fitness and cognitive capacity were not assessed in this study. Physical fitness has been identified as an important moderator of cognitive outcomes following acute exercise, with greater benefits often observed in individuals with lower fitness levels (Ishihara et al., 2021). Conversely, other research has suggested that individual differences in cognitive reserve, rather than physical fitness, may better account for variability in exercise-induced enhancements in executive function (Ludyga et al., 2016). Therefore, future studies should include baseline assessments of both physical and cognitive capability to clarify the factors contributing to variability in the acute effects of cognitively demanding exercise on executive function.

Fourth, heart rate data were available for only 14 participants who agreed to wear the monitor. Although the heart rate was recorded from the same participants across all three interventions, this subsample may not fully represent the entire sample. This limits confidence in the exercise intensity manipulation check. Moreover, ratings of perceived exertion suggested that exercise intensity fell within the intended range (HE: 7.3; LE: 7.2), whereas heart rate values indicated a higher-than-intended range (HE: 82.7% HRmax; LE: 83.0% HRmax). The findings should therefore be interpreted in the context of higher-than-intended physiological intensity. Future studies should collect heart rate data from the full sample and adjust exercise intensity in real time to maintain the intended range.

Fifth, the manipulation check relied solely on MERS ratings to differentiate cognitive demand between the exercise conditions. Objective performance indicators during the exercise tasks were not recorded. Although MERS scores were significantly higher in HE than in LE, the mean score was 4.0 on the 9-point scale. This indicates that the absolute level of perceived mental effort in HE was relatively modest. Therefore, the HE and LE labels should be interpreted as a relative difference in cognitive demand rather than as indicating absolute levels of task difficulty. Additionally, the fixed task difficulty may not have posed an equivalent level of challenge across individuals. Future studies could include objective performance indicators (task accuracy, error rates, response times) and adopt adaptive task designs that dynamically adjust cognitive load based on participants’ ongoing performance (Anzeneder et al., 2023a). These approaches may help clarify the dose–response relationship between cognitive demand during exercise and cognitive outcomes (Jäger et al., 2015).

Sixth, although an interval of at least 72 h was maintained between experimental sessions to minimize potential carryover effects across conditions, residual carryover effects on cognitive outcomes cannot be ruled out. Additionally, information regarding intervention sequence and study period was not retained; therefore, potential period and sequence effects could not be evaluated retrospectively. Future crossover studies should retain and incorporate period and sequence information to formally account for these potential effects.

Seventh, the CAF and DP analyses were added after data collection as exploratory secondary analyses and were not included in the original sample-size determination. With 26 participants, these complex distributional and interaction analyses may have had limited power to detect small or moderate condition-related effects. In addition, because these analyses were based only on post-test data collected after each intervention, condition-specific baseline variability could not be fully accounted for when comparing conditions.

Finally, data collection was affected by the COVID-19 pandemic. Some of the participants experienced up to 3-week delays between intervention sessions due to the school closures. Although these disruptions were unavoidable, they may have introduced additional variability in the timing of the interventions and consequently in the observed effects. Nevertheless, the study was completed successfully, demonstrating the feasibility of conducting controlled experimental research under real-world educational constraints.

## Data Availability

The raw data supporting the conclusions of this article will be made available by the authors, without undue reservation.
