# Supplementary material for: Effects of single bout exercise with different cognitive demands on inhibitory control in elementary school children
Source: Front Psychol. 2026 Oct 7;17:1879513. doi: 10.3389/fpsyg.2026.1879513 (PMC13683003; doi:10.3389/fpsyg.2026.1879513)
Supplement: Supplementary file 1 [file Table_1.docx]

**Baseline comparability of cognitive performance**

Given that potential imbalance in the cognitive performance across conditions (see Supplementary) could influence the interpretation of the outcomes, four additional one-way repeated-measures ANOVAs were conducted to compare pre-intervention reaction time, and accuracy, separately for congruent and incongruent trials.

The results suggested that there were no significant differences across conditions for congruent reaction time, *F* (1.44, 35.94) = 1.53, *p* = .231, $\eta_{p}^{2}$= .058, incongruent reaction time, *F* (1.51, 37.67) = 2.03, *p* = .156, $\eta_{p}^{2}$= .075, congruent accuracy, *F* (1.10, 27.45) = 1.87, *p* = .183, $\eta_{p}^{2}$= .070, and incongruent accuracy, *F* (1.34, 33.45) = 0.42, *p* = .578, $\eta_{p}^{2}$= .017.

**Decomposition of the Flanker effect**

To clarify the condition-related difference in reaction time Flanker effect, additional 3 (Condition: HE, LE, AC) x 2 (Time: pre, post) repeated-measures ANOVAs were conducted separately for congruent and incongruent trials using.

For the congruent reaction time, a significant Condition x Time interaction was observed, *F* (1.50, 37.35) = 5.72, *p* = .012, $\eta_{p}^{2}$= 0.19. Post hoc pairwise comparisons indicated that participants responded significantly faster in LE than in AC at post test, *t* (25) = -5.01, *p* < 0.001, *d* = -0.78, 95% CI [-1.41, -0.16]. The corresponding pre-to-post changes were -19.6 ms in the HE, -8.7 in the LE and +19.5 in the AC.

For the incongruent reaction time, the Condition x Time interaction was not significant. However, a significant main effect of Time was observed, *F* (1, 24) = 32.72, *p* < .001, $\eta_{p}^{2}$= 0.58, indicating that incongruent reaction times were shorter at post-test than at pre-test across conditions. Descriptively, incongruent reaction time decreased by 41.7 ms in HE, 21.6 ms in LE, and 25.8 ms in AC.

*Conditional accuracy function in congruent trials among three conditions.*

*Note.* HE = high cognitive-demanding exercise; LE = low cognitive-demanding exercise; AC = active control; (error bars show standard errors).
